# Evaluating the impact of land use/cover changes on hydrological processes in the Lake Tana Basin

**DOI:** 10.1038/s41598-025-26817-0

**Published:** 2025-11-21

**Authors:** Eman M. Ragab, Amr Fleifle, Nesma Abdelmeged, Mohammad Abourohiem

**Affiliations:** Irrigation and Hydraulics Department, Faculty of Engineering, Alexandria University, Alexandria, Egypt

**Keywords:** Lake Tana Basin, SWAT, Remote sensing, Hydrological processes, Land use/cover, Hydrology, Hydrology

## Abstract

Land use/cover (LULC) changes has a fundamental effect on the hydrological components in the Lake Tana Basin. The Lake Tana Basin, the source origin of the Blue Nile, has experienced notable LULC transitions over the past two decades. The present study evaluates the effect of land use/cover (LULC) changes on hydrological components in the Lake Tana basin using the soil and water assessment tool (SWAT). Two LULC maps, one from the International Livestock Research Institute (ILRI) for 2004 and another developed from Landsat 8 images for 2021, were used. Both models were calibrated and validated by SUFI-2 using observed discharge data, results showed strong performance (NSE > 0.79, R^2^ > 0.79 calibration; NSE > 0.90, R^2^ > 0.94 validation). Between 2004 and 2021, agricultural land decreased by 10.2% and forest cover declined by 33.1%, while wetlands and rangelands increased by 81.4 and 299.2%, respectively. Moreover, urban land was presented as a new class. These changes affected the basin’s hydrology as surface runoff increased from 111.6 to 118 mm/year (+ 5.8%), lateral flow decreased from 106.3 to 100.7 mm/year, and shallow aquifer evaporation declined by 10.2%. Evapotranspiration remained nearly constant at 1066 mm/year dominated by the lake evaporation. The results confirm the significant influence of LULC changes on the hydrological components of the Lake Tana Basin which highlight the need for sustainable land and water management.

## Introduction

Land use/cover (LULC) change is a primary driver of hydrological variability in the Upper Blue Nile. The Lake Tana Basin, the headwater source of the Blue Nile, has exposed to LULC changes all over the recent decades which affected the hydrological process of the basin. Understanding such effect is essential for basin planning and water security. The present work focuses on isolating the hydrologic effects of LULC by comparing two time-specific LULC datasets (2004 vs. 2021). Such a comparison was conducted under consistent climate forcing using the SWAT model, and quantified the LULC changes on water-balance components in the Lake Tana Basin.

Some previous studies are presented to analyse the water balance for the Blue Nile specially Lake Tana basin. Kebede et al.^1^ studied the water balance in Lake Tana and its sensitivity to fluctuations in rainfall and the results revealed that the lake is relatively insensitive to rainfall variations. Stegan et al.^2^ applied a SWAT model for the Lake Tana Basin which showed that baseflow about 40% − 60% and more than 60% of the rainfall is lost in the watershed due to evapotranspiration.

Other studies concentrated on climate change impacts. Gebreyohannes et al.​^3^ investigated the potential impacts of climate change on the hydrological regime of the Lake Tana Basin and the results projected temperature increases of up to 4.4 °C, while precipitation trends varied by scenario and sub-basin. Wubneh et al.^4^ assessed the future operational performance of Lake Tana under climate change conditions by integrating HBV hydrological modeling with the HEC-ResSim reservoir simulation tool.

A third group of studies examined LULC change impacts. Mekonnen et al.^5^ analyzed the effects of land use and land cover (LULC) changes and climate change on streamflow for the whole Upper Blue Nile River basin. Kidane et al.^6^ constructed a hydrological model via SWAT software to study the response of climate and land use/land cover (LULC) dynamics on the Guder River which is a tributary of the Upper Blue Nile (Abbay) River. Getachew et al.^7^ applied the SWAT + model to assess the hydrological impacts of land use/cover (LULC) changes in the Lake Tana Basin over a 30-year period. Utilizing Landsat images from 1989, 2005, and 2019. The study documented extensive conversion of shrublands, grasslands, and bare land into farmlands and built-up areas. Hydrological simulations revealed that these LULC changes led to increased surface runoff and water yield, while evapotranspiration and baseflow decreased. Engdaw et al.^8^ examined the impacts of land use/land cover (LU/LC) changes on water quality in the northern watershed of Lake Tana over a 30-year period (1993–2022) using Landsat imagery and supervised classification techniques. The analysis revealed a marked decline in cropland (from 71.4% to 38.7%) and an increase in settlement, forest, and grassland cover.

The above studies revealed the research gap. In which an advance knowledge on both climate and land-use impacts were conducted as a combined effect. Very few studies separate the hydrologic effect of LULC by fixing climate inputs. Then, quantify component-wise responses (runoff, lateral flow, groundwater exchanges, ET) at basin scale focusing on Lake Tana basin, and test how LULC map source and spatial resolution influence SWAT outputs.

The objective of the current research is to isolates and quantifies the hydrologic impacts of LULC changes in the Lake Tana Basin by comparing two LULC datasets, ILRI (2004) and a newly classified Landsat 8 map (2021), under consistent climate in the SWAT model. Specifically, the study (i) evaluates the magnitude of LULC change between 2004 and 2021, (ii) quantifies its impacts on water balance components, and (iii) highlights implications for sustainable water and land management in the basin.

## Methods and materials

### Study area

**Fig. 1:**
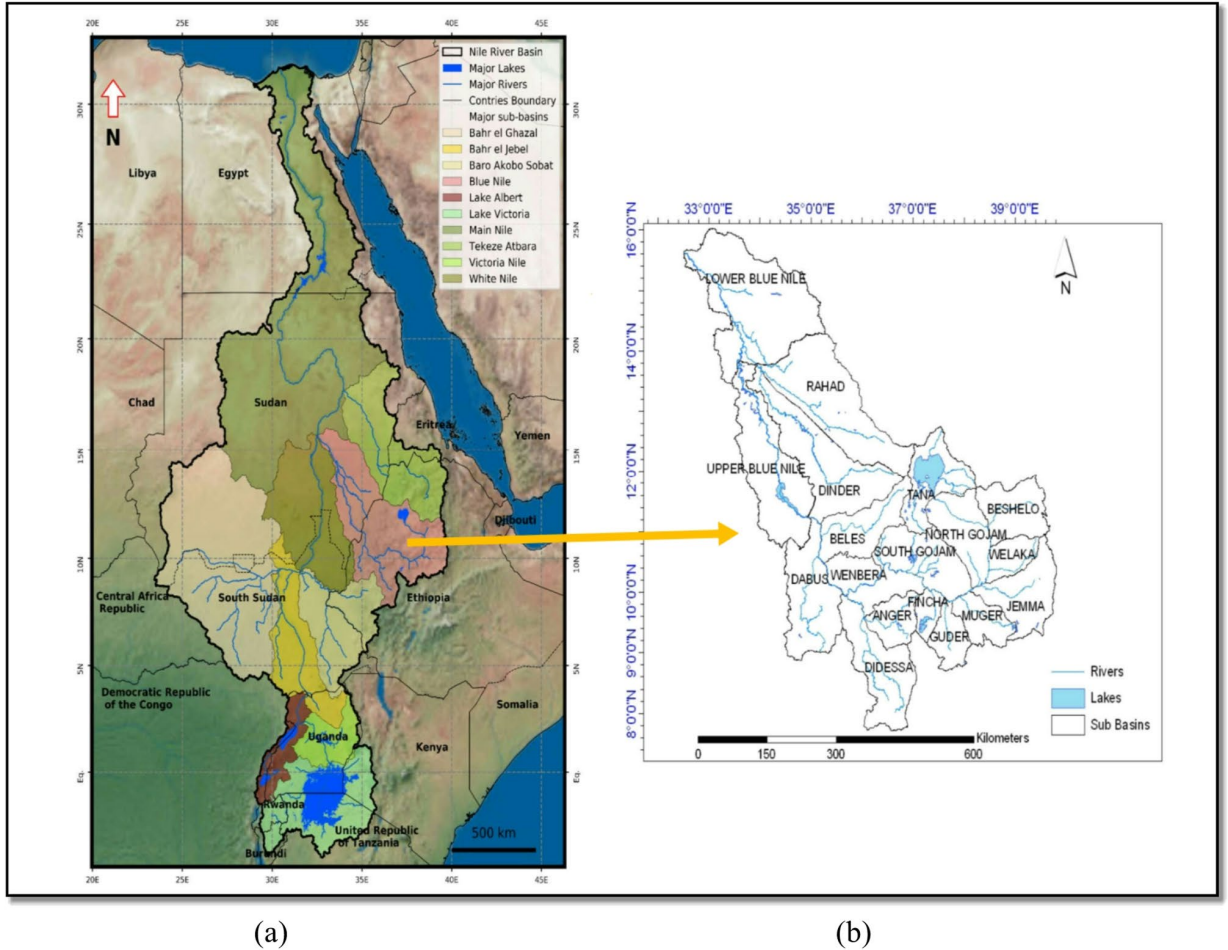
(**a**) Location of the Nile River Basin and its major subbasins^9^. (**b**) The Blue Nile subbasin^11^. Both maps are adapted from the cited sources and arranged together by the author for illustrative purposes.

Three main sources contribute to the water volume in the Nile River; the western Nile River from Bahr El Ghazal, the southern Nile River from Bahr El Jebel, and the eastern Nile River from the Ethiopian Plateau as shown in (Fig. 1a)^9^. The Nile basin covers about 3.1 million km², which represents approximately 10% of the African continent. The Ethiopian highlands provide 86% of the Nile flow; 59% from the Blue Nile, 14% from BaroAkobo Sobat, and 13% from Atbara^10^. The Blue Nile River flows from the Ethiopian Plateau at elevations greater than 4000 m above sea level (m a.s.l.) to an elevation of approximately 350 m (a.sl.) in Khartoum^2^. The Blue Nile is divided into 18 subbasins, as shown in (Fig. 1b)^11^.

**Fig. 2:**
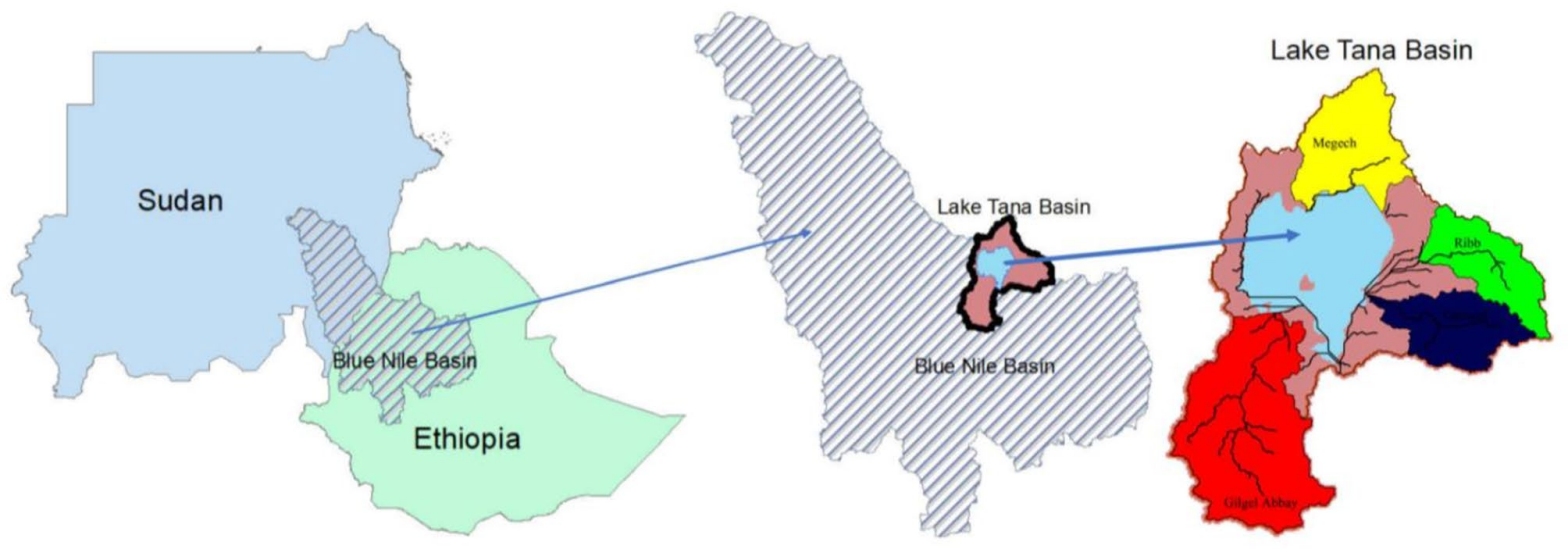
Locations of the study area and the Gilgel Abbay, Gumera, Ribb, and Megech subbasins. The map was created by the author using ArcGIS 10.1 (ESRI, https://www.esri.com).

Lake Tana Basin, located in the Ethiopian highlands, is the primary source of the Blue Nile, and has a significant importance to the hydrology the basin. Lake Tana is Ethiopia’s largest freshwater lake, located in the center of the basin. The area of the Lake Tana basin is about 15,000 km^2^, and located between latitudes 10˚ 55′ to 12˚ 45′ N and longitudes 36˚40′ to 38˚ 20′ E. Lake Tana has a surface area of approximately 3000 km^2^^10^. The lake is about 84 km long and 66 km wide, with an average depth of about 8 m and a maximum depth of 15 m, which make it a shallow lake and results in high evapotranspiration rates between 1,700 and 2,200 mm per year^1^. The basin includes more than 40 torrents that discharge into Lake Tana, but Gilgel Abbay, Gumera, Ribb, and Megech contribute more than 93% of the inflow into the lake as shown in Fig. 2. The catchment area rises up to 4000 m above sea level (a.s.l.) in the north of the basin, but the lake is located at an elevation of 1800 m (a.s.l.). Rainfall in the basin varies between 800 mm and 2200 mm annually according to elevation and location. The basin has one rainy season between June and September.

### Dataset

#### Land use/cover preparing and processing (LULC-2021)

Land use and land cover (LULC) mapping is a fundamental step in hydrological modeling, as it defines the spatial distributions of different land uses and human activities. In this study, the LULC map for the year 2021 was developed by using remote sensing techniques and processes using ERDAS Imagine software. To cover the extensive study area, three different satellite images were downloaded from the United States Geological Survey (USGS) Earth Explorer website^12^ for Landsat-8 Operational Land Imager (OLI) for May 2021 (dry season), and the images were selected considering the minimal cloud cover to ensure maximum clarity and visibility.

**Fig. 3:**
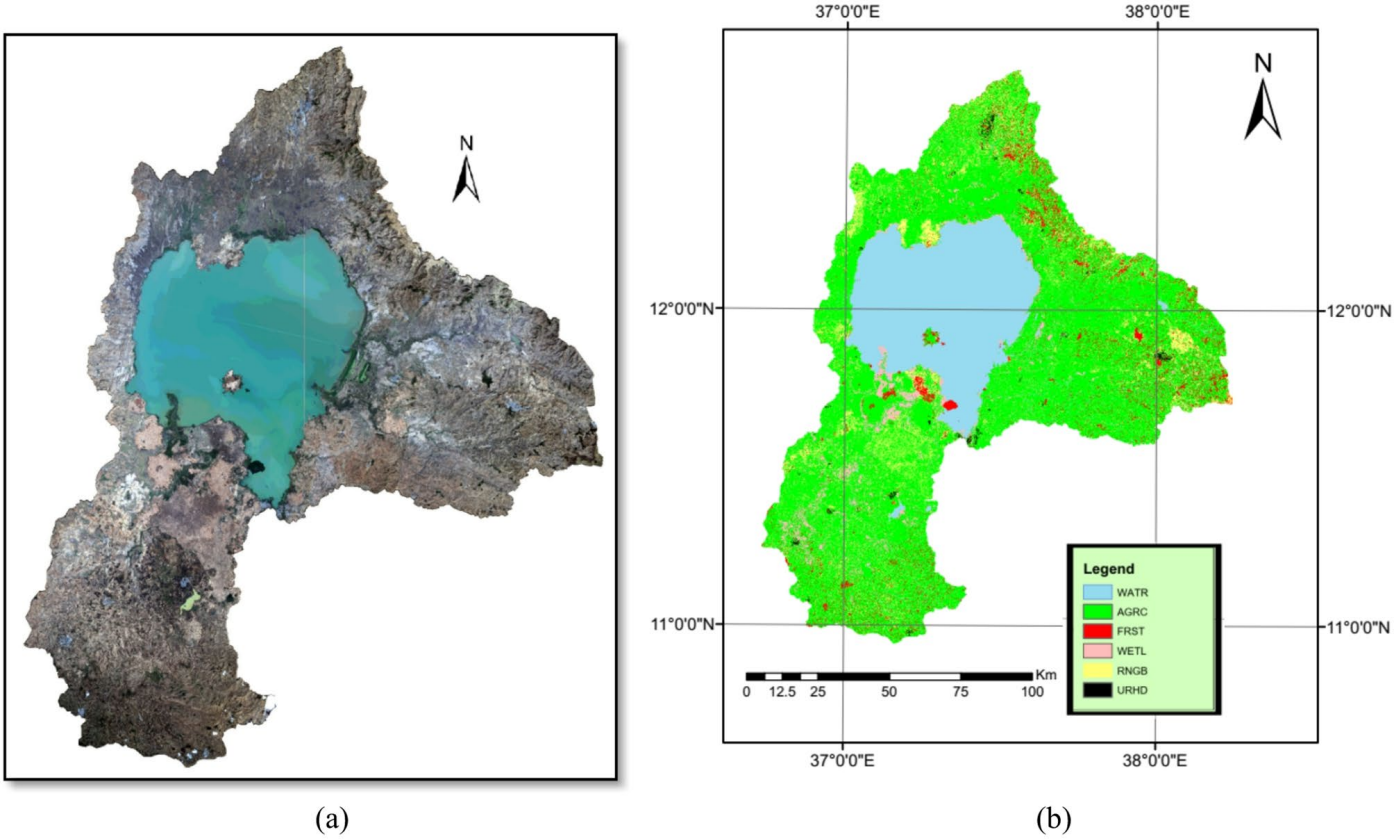
(**a**) Landsat 8 satellite images before classification. (**b**) Classified LULC-2021 map. Both maps were processed and generated by the author using ERDAS Imagine 2015 (Hexagon Geospatial, https://www.hexagongeospatial.com).

Then, the images were processed by layer stacking to create multispectral composite images. Landsat 8 images consisted of multiple spectral bands; only bands 1,2,3,4,5,6, and 7 (coastal / blue / green / red / NIR / SWIR1 / SWIR2) were used in the layer stacking step to create multispectral composites. The composite images were processed by mosaicking to create one image to cover the entire Lake Tana Basin, as shown in (Fig. 3a).

A combined (hybrid) classification approach was then applied to enhance the accuracy of land cover mapping. First, an unsupervised classification grouped the imagery into 200 spectral classes. Next, ERDAS was geographically linked with Google Earth to perform visual cross-checks, after which clusters were re-labeled into six final LULC classes based on spectral/visual evidence: water (WATR), agriculture (AGRC), forest (FRST), wetland (WETL), rangeland (RNGB), and urban land (URHD)). The final LULC-2021 map (Fig. 3) thus integrates the strengths of both unsupervised clustering and supervised interpretation. as shown in Fig. 3b. Finally, the map was revised using ERDAS 2014 accuracy assessment tool and Google Earth software.

Accuracy assessment is very fundamental in LULC classification. The classified LULC need to be approved for reliability and quality of the classified map. The map needs to be validated to ensure the map does not mislead the conclusions and the results. The map will be used to construct a SWAT model that will be used to analysis effect of land use change on the hydrological balance. Accuracy assessment evaluate if the classification represents the actual LULC by comparing the classified data with the ground data. Statistical metrics such as overall accuracy, Cohen’s kappa coefficient, and user’s and producer’s accuracy gives general and Classwise performance perspective. A total of 90 random points were generated to calculate the classification accuracy of the classified LULC-2021 map via ERDAS, and each point was investigated via Google Earth to complete the accuracy assessment table. The results of the accuracy assessment are as shown below:

##### Overall classification accuracy

The LULC classification achieved an accuracy of 91.11% which is considered high accuracy and represents strong agreement between the classified map and the ground data. The percentage confirms the effectiveness of the classification methodology used in processing the map.

##### Overall kappa statistics

The Cohen’s kappa coefficient for the classification achieved was 0.8937, which reflects “almost perfect agreement” according to standard interpretation scales^13^. A kappa value above 0.80 indicates that the classification is highly reliable.

##### Classwise accuracy analysis

For the separate classes the product’s and user’s accuracy percentages and the kappa coefficient are presented in Table 1. All classes show strong performance, especially Classes 1, 3, and 6 with perfect classification.

**Table 1:** Class accuracy analysis.

Class	product’s accuracy percentage (%)	user’s accuracy percentage (%)	Kappa coefficient	
Water	100	100	1	Perfect classification
Agriculture	77.78	93.33	0.9167	Excellent classification
Forest	93.33	93.33	0.92	Excellent classification
Wetland	90.91	71.43	0.6745	Good classification
Range land	92.86	86.67	0.8421	Very good classification
Urban land	100	100	1	Perfect classification

#### Digital elevation model (DEM)

**Fig. 4:**
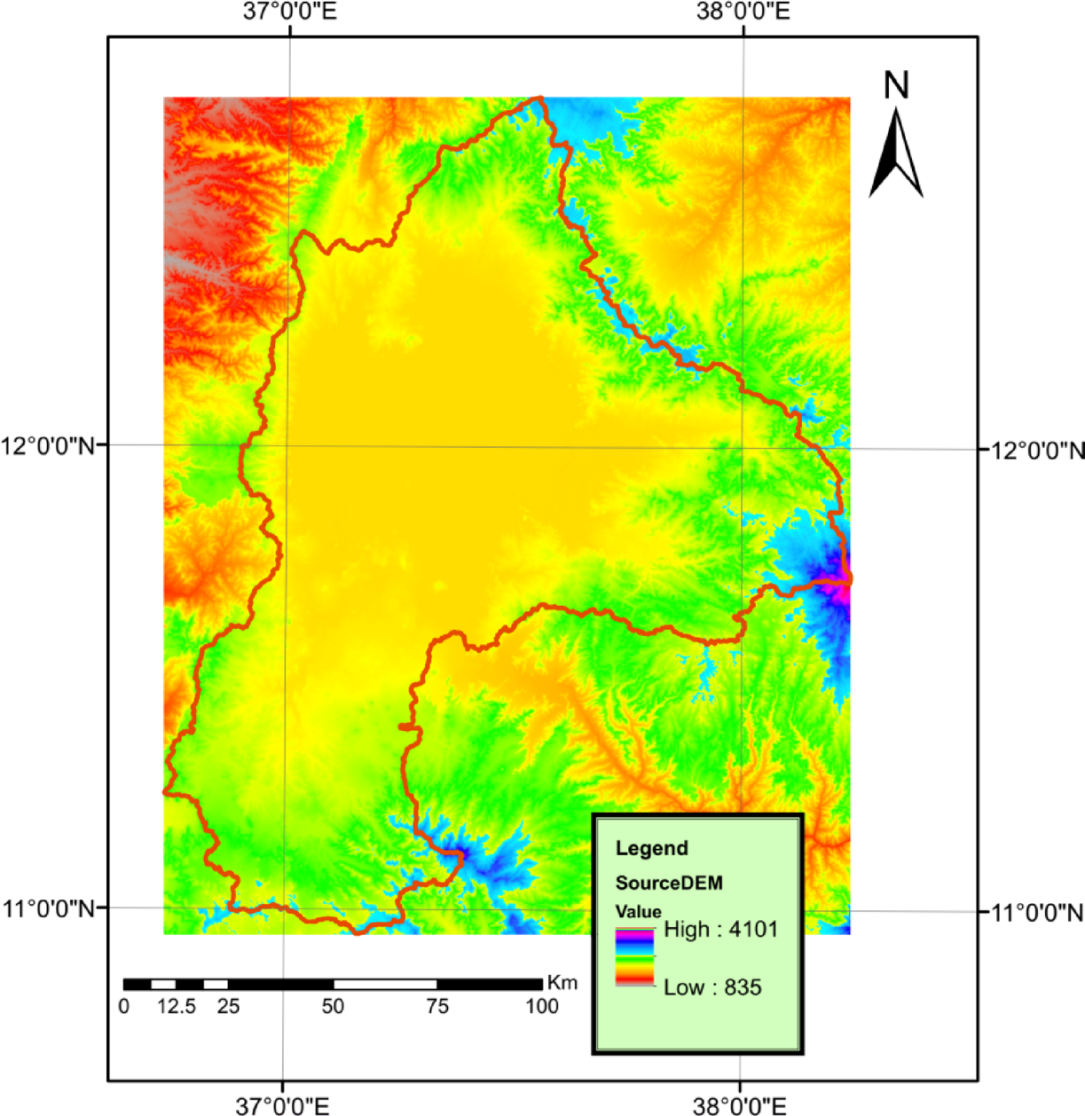
Digital elevation model for the Lake Tana Basin and the delineated streams. The map was created by the author using ArcGIS 10.1 (ESRI, https://www.esri.com).

The Digital Elevation Model (DEM) map was produced by the Advanced Spaceborne Thermal Emission and Reflection Radiometer (ASTER) Global Digital Elevation Model (GDEM) V003. The ASTER GDEM covers land surfaces between latitudes 83°N and 83°S. The DEM map resolution is 1 arc-second (30*30 m). The map was obtained from the NASA website^14^. The DEM was used to delineate the basin and generate the stream network shown in Fig. 4.

#### Soil data

**Fig. 5:**
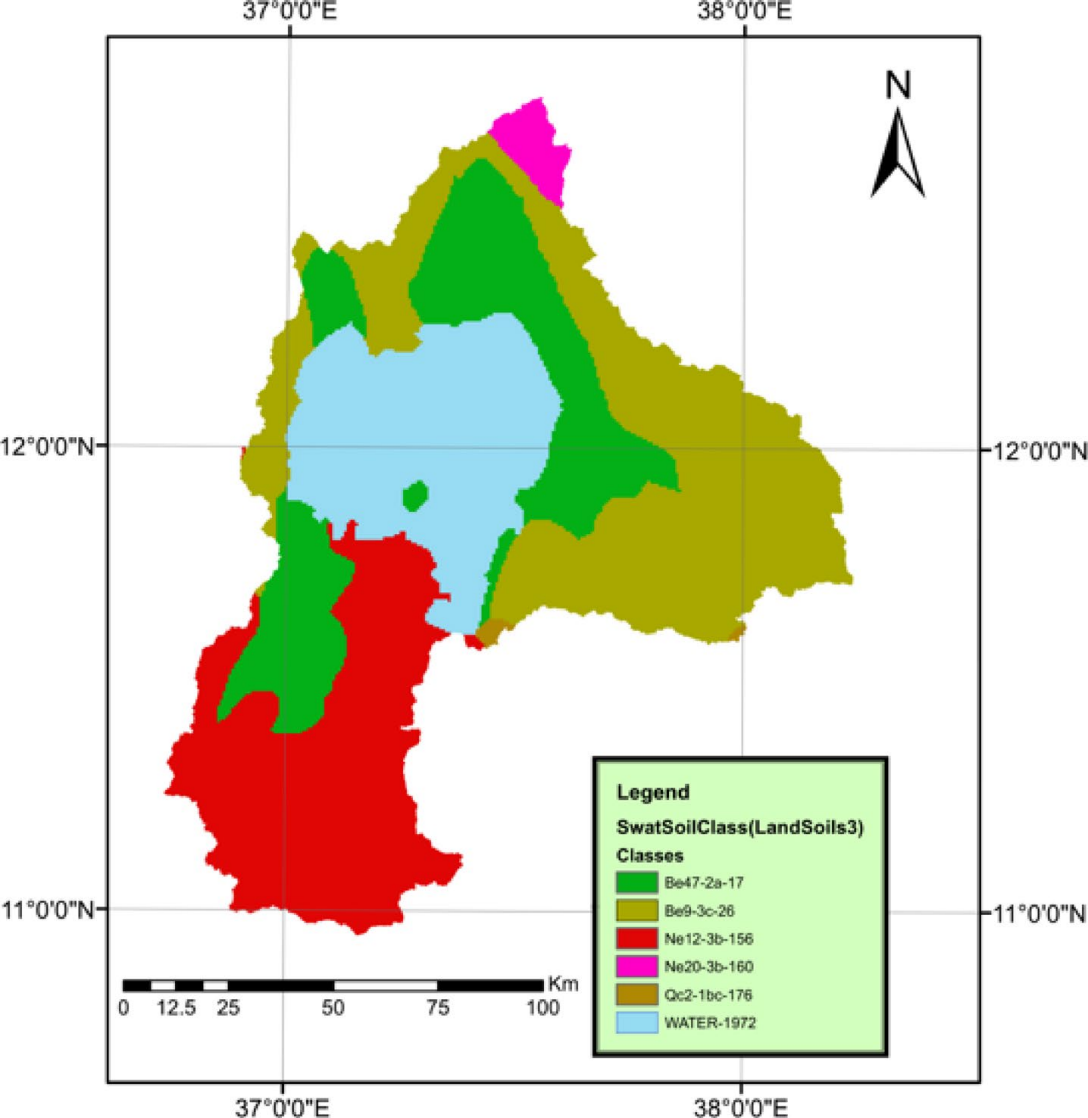
FAO soil map of the study area. The base soil data were obtained from the FAO Harmonized World Soil Database (https://www.fao.org/soils-portal/data-hub/soil-maps-and-databases/en/). The map for the Lake Tana Basin was prepared by the author using ArcGIS 10.1 (ESRI, https://www.esri.com).

The soil map was obtained from the Food and Agriculture Organization (FAO) of the United Nations website at a scale of 1:5,000,000. The Harmonized World Soil Database, a raster dataset with a spatial resolution of 30 arc-seconds, integrates over 15,000 distinct soil mapping units, combining regional and national soil data updates worldwide as shown in Fig. 5. The FAO soil map is available through the FAO site^15^. The soil map classes were divided into 6 classes where the (Be) class was Eutric Cambisols, the (Ne) class was Eutric Nitosols, the (QC) class was Cambic Arenosols, and water.

#### Weather data

Weather data were obtained from The Prediction of Worldwide Energy Resources (POWER) which is available through the NASA website^16^. All datasets were generated from numerical weather prediction models between 1984 and 2021. NASA POWER provides meteorological data including temperature, relative humidity, precipitation, solar radiation, and wind.

#### Land use map

**Fig. 6:**
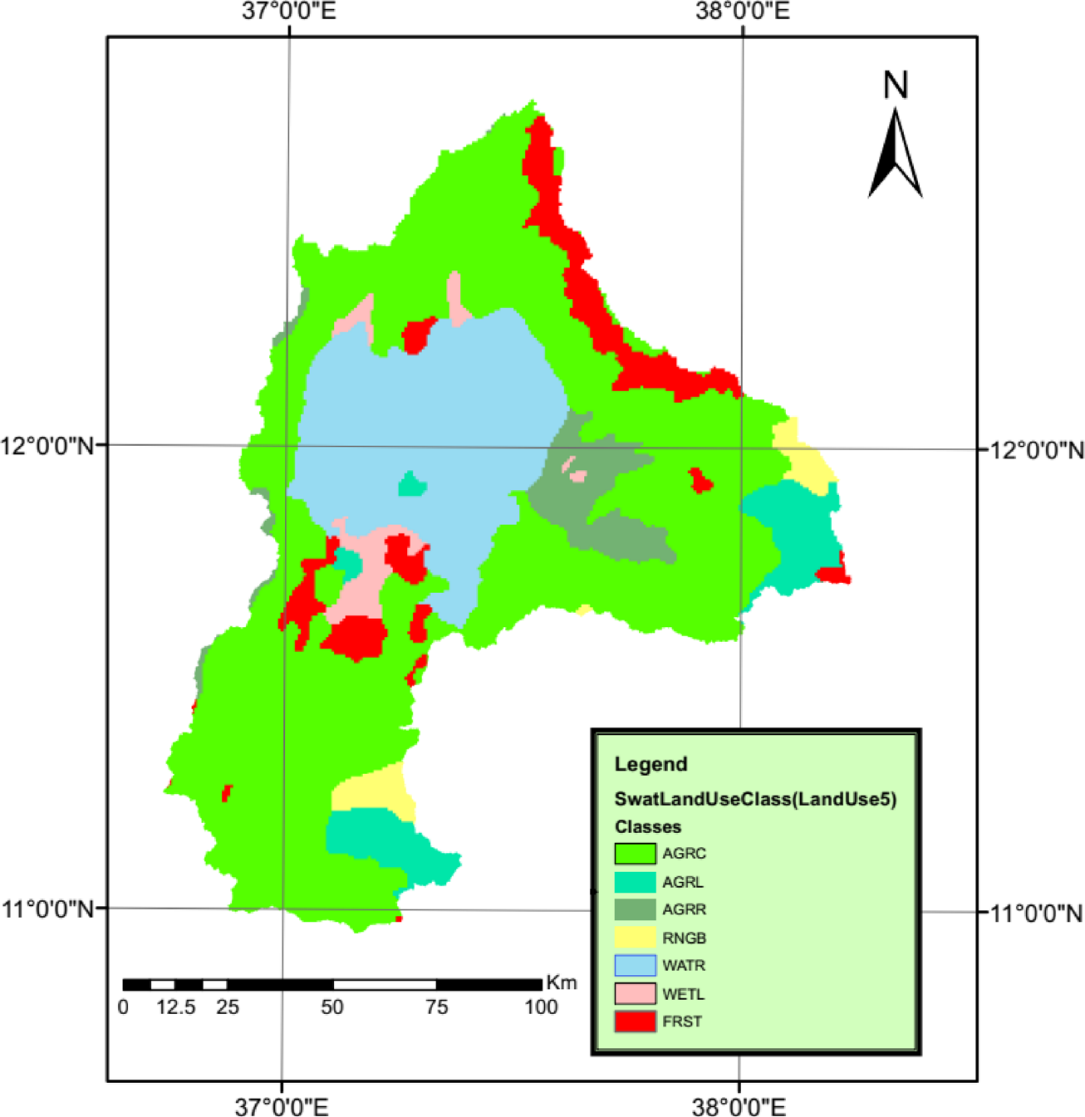
ILRI land use/land cover (LULC) map of 2004 for the Lake Tana Basin. The original data were obtained from the International Livestock Research Institute (ILRI) GeoPortal (https://data.ilri.org/portal/). The map for the Lake Tana Basin was prepared by the author using ArcGIS 10.1 (ESRI, https://www.esri.com).

Two land use maps were considered to construct two swat models, which are detailed below;

(I) The first map was obtained from the GIS portal of the International Livestock Research Institute (ILRI) for the year 2004^17^ and has a resolution of 670*670 m as shown in Fig. 6.
(II) The second map was developed by using remote sensing techniques and was processes using Erdas Imagine software as previously described.

A comparison was conducted between the two maps to clarify the change in land use/cover as shown in Table 2. The ILRI LULC-2004 neglected urban areas, whereas the new map considered urban land use. The forest area decreased by 33%, and the agricultural area decreased by 10.17%. Rangelands and wetlands expanded significantly, with increases of 299.20% and 81.44%, respectively. There was an increase in water bodies of 7.1%. some of the observed changes, particularly the large increase in rangeland (+ 299.2%), may partly reflect differences in classification methodology, spatial resolution, and class definitions between the ILRI-2004 and the 2021 Landsat-based LULC maps, in addition to genuine land use dynamics. All these changes reflected both LULC changes and differences due to classification accuracy. Unlike the newly classified 2021 map, the ILRI LULC-2004 dataset does not provide a documented accuracy assessment, which introduces some uncertainty when comparing the two maps.

**Table 2:** A comparison between the area of land use in both ILRI LULC-2004 and classified LULC-2021.

	ILRI LULC-2004 Area Km^2^	Classified LULC-2021 Area Km^2^	% Change
Water	2981.086	3192.69	7.10%
Agriculture	10,244	9203.16	-10.17%
Forest	1081.408	724.383	-33.06
Wetland	340.44	617.68	+ 81.44%
Range land	322.385	1286.804	+ 299.20
Urban land	–	68.338	New class
Total area	14969.478	15093.056	

#### Discharge data

The discharge data used for calibration and validation were obtained from the Ministry of Water and Energy in Ethiopia^18^. The monthly discharge data were gathered from the Addis Zemen gauging station. For calibration the discharge data between 1992 and 1996 were used. The data for the years 2004 and 2005 were used for validation.

### SWAT model setup

The present study applies the physically based watershed model SWAT to simulate the hydrological process in the Lake Tana basin. The purpose of this study is to investigate the basin water balance under the influence of topographic, soil, and weather data but to focus mainly on the effects of land use/cover changes on the water balance components. Therefore, the application of the model involved calibration, uncertainty analysis, sensitivity analysis, and validation using Sequential Uncertainty Fitting algorithm (SUFI-2) within the SWAT-CUP program. SWAT uses spatial data to simulate water at the catchment scale on yearly, monthly, or daily scales. SWAT uses Hydraulic Response Units (HRUs) that divide the area according to land use, soil and slope. The program calculates the water balance component on the basis of the water balance equation represented in Eq. (1)^19^.

1 $$SW_{t} = SW_{0} + \sum\nolimits_{{i = 1}}^{t} {\left( {{\text{R}}_{{{\text{day}}}} - {\text{Q}}_{{{\text{surf}}}} - {\text{E}}_{{\text{a}}} - {\text{w}}_{{{\text{seep}}}} - {\text{Q}}_{{{\text{qw}}}} } \right)}$$

where;

SW_t_ : Final soil water content (mm).

SW_0_: Initial soil water content on day i (mm).

t: Time (days).

R_day_: Precipitation on day i (mm).

Q_surf_: Surface runoff on day i (mm).

E_a_: Evapotranspiration on day i (mm).

w_seep_: Water entering the vadose zone from the soil profile on day i (mm).

Q_qw_: Return flow on day i (mm).

### Watershed delineation

**Fig. 7:**
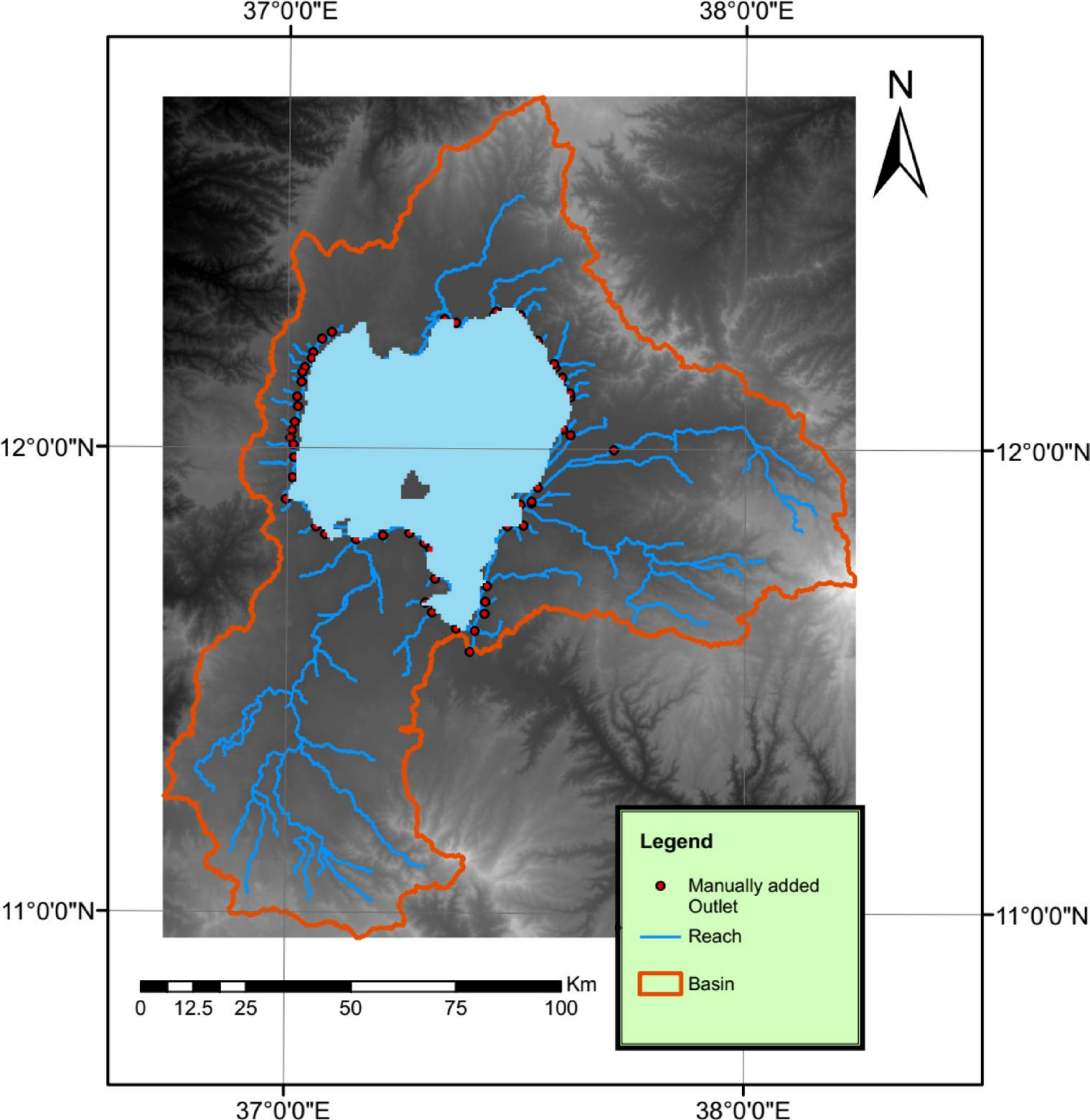
Watershed delineation and reach generation. The map was generated by the author using ArcSWAT (SWAT extension for ArcGIS 10.1; https://swat.tamu.edu/software/ar).

SWAT was used to simulate two models for the study area. First, the DEM was used to generate the reaches and delineate the 54 subbasins for both models as shown in Fig. 7. Second, for the first model, using the ILRI LULC-2004 and the soil map a total of 172 HRUs were created. For the second model which uses the processed classified LULC-2021 map a total of 278 HRUs were created. For both models, thresholds of 10%, 20% and 10% were assigned for land use, soil and slope respectively. Then the weather data were input into both SWAT models. The models were executed with a monthly time step. For both models, all the inputs were the same except for the LULC map, which was used to study the effects of the land use/cover changes on all the water balance components. The simulations were performed between 1984 and 2021, and a warm-up period of 3 years was used to reach acceptable values for the model parameters. After that, the models were calibrated and validated via the SWAT-CUP program using the SUFI-2 algorithm and a sensitivity analysis was conducted. Finally, the fitted parameters satisfying the statistical indicators were used to manually calibrate the SWAT models.

## Results and discussion

### Sensitivity analysis

Sensitivity analyses were performed for two different land use/cover scenarios: the classified LULC-2021 and the ILRI LULC-2004. The results are illustrated in Table 3. For both scenarios identified as CN2, GWQMN and ALPHA_BNK were the most sensitive parameters, as indicated by their very low p-values (*p* < 0.05). However, the classified LULC-2021 was more sensitive for GWQMN with a p-value of 0.0010, whereas the p-value for the ILRI LULC-2004 was 0.0406. For the classified LULC-2021, SOL_K and EPCO indicated moderate sensitivity, whereas ALPHA_BF had lower sensitivity. Parameters such as the groundwater delay (GW_DELAY), groundwater evaporation coefficient (GW_REVAP), channel roughness (CH_N2), and hydraulic conductivity (CH_K2) displayed lower sensitivities. Similarly, for the ILRI LULC-2004, parameters such as ALPHA_BF, SOL_K, and EPCO were moderately sensitive, whereas GW_REVAP, CH_N2, GW_DELAY, and SOL_AWC demonstrated relatively low sensitivity. In both scenarios, the maximum canopy storage (CANMX) and channel hydraulic conductivity (CH_K2) have the least sensitive parameters. The sensitivity analyses indicate the huge influence of surface runoff and shallow aquifer in the results of hydrological process, showing the importance of CN2, GWQMN, and ALPHA_BNK across different LULC configurations. The sensitivity of the parameters indicates that the classified LULC-2021 increased sensitivity to parameters that regulate soil infiltration and plant water uptake, because of more detailed or more realistic representations of vegetation and land cover. The ILRI LULC-2004 was less influenced by soil and plant processes, which may indicate a coarser classification or a large land cover cell size. This finding emphasis that higher-resolution LULC datasets, such as the 2021 map, enhance the model’s sensitivity to soil–plant–water interactions, which provide more reliable insights into hydrological processes compared to coarser, generalized maps.

**Table 3:** Sensitivity analysis for the parameters t-stat and p-value.

Parameters	ILRI LULC-2004		Classified LULC-2021	
	t-stat	*p*-value	t-stat	*p*-value
CANMX.hru	-0.207826774	0.835451107	-0.182405091	0.855340735
SOL_AWC(.).sol	0.212577195	0.831745709	0.268721585	0.788257738
CH_K2.rte	-0.248900488	0.803542690	-0.089710171	0.928554415
GW_DELAY.gw	-0.572872403	0.566995487	-0.706445552	0.480248940
GW_REVAP.gw	0.696921848	0.486184233	0.828482599	0.407803082
CH_N2.rte	0.843391165	0.399423762	0.994501602	0.320472593
ALPHA_BF.gw	-1.354702461	0.176140494	-1.327113687	0.185092927
EPCO.hru	1.865461746	0.062717777	1.171694846	0.241892501
SOL_K(.).sol	-1.886949510	0.059761642	-1.495757497	0.135364589
ALPHA_BNK.rte	-2.576649885	0.010269625	-2.495839329	0.012895669
GWQMN.gw	3.306062316	0.001016029	2.053187118	0.040588039
CN2.mgt	-25.013173413	0.000000000	-25.074297072	0.000000000

### Calibration and validation

A set of 12 parameters was chosen for calibration of the SWAT model. These values were modified to estimate parameters by multiplying, replacing, or adding values. The description and range of parameters used in the SWAT calibration are given in Table 4. The 12 parameters selected include: GW_REVAP controls the movement of water from the shallow aquifer to the unsaturated zone. CH_N2 is the Manning’s roughness coefficient for the main channel that influences flow velocity and sediment transport. CH_K2 represents the effective hydraulic conductivity in the main channel alluvium. ALPHA_BNK is the baseflow alpha factor for bank storage that determines the response time of bank flow. SOL_AWC is the available water capacity of the first soil layer. SOL_K is the saturated hydraulic conductivity of the first soil layer. CN2 is the SCS runoff curve number that affects surface runoff estimation. ALPHA_BF is the baseflow alpha factor that indicates the baseflow response to changes in recharge. GW_DELAY is the groundwater delay time that simulates the lag between water recharge and the baseflow contribution. GWQMN is the threshold depth of water in the shallow aquifer required for return flow to occur. CANMX refers to the maximum canopy storage that indicates the vegetation’s capacity to intercept rainfall. EPCO is the plant uptake compensation factor that regulates the extent to which plants can take water from different soil layers.

**Table 4:** Parameters used for calibration and validation in each scenario.

parameter	Type of change	ILRI LULC-2004			Classified LULC-2021		
		Fitted value	min	max	Fitted value	min	max
GW_REVAP.gw	r	0.0959	-0.1	0.2	-0.0199	-0.1	0.2
CH_N2.rte	v	0.22475	0.2	0.45	0.2387	0.2	0.5
CH_K2.rte	v	54.5	30	130	51.099998	30	130
ALPHA_BNK.rte	v	0.106	0	0.4	0.0804	0	0.4
SOL_AWC.sol	r	0.30215	0.1	0.75	0.26625	0	0.75
SOL_K.sol	r	0.7672	0	0.8	0.4616	0	0.8
CN2.mgt	r	-0.1011	-0.15	0.15	-0.0939	-0.15	0.15
ALPHA_BF.gw	r	0.173	-0.5	0.5	0.5747	-0.5	0.6
GW_DELAY.gw	v	433.850006	150	500	238.550003	150	500
GWQMN.gw	v	4377.5	2500	5000	4739	2000	5000
CANMX.hru	r	0.5817	0	0.7	0.741	0	1
EPCO.hru	v	0.7345	0.3	0.8	0.785	0.2	0.8

The parameters with the type of change (r) were adjusted relative to the existing values via the formula (1 + fitted value). The parameters with the type of change (v) replaced the existing values. All fitted parameters remained within physically acceptable and model-recommended limits.

Both SWAT models were calibrated and validated using SUFI-2 in the SWAT-CUP program. Streamflow at the FLOW_OUT_29 subbasin (Ribb Basin) discharge was used between 1992 and 1996 for calibration and between 2004 and 2005 for validation. Five hundred simulations were performed for both models. Both models yield satisfactory results as shown in Table 5. Both models’ calibration results indicate excellent model performance. The models’ uncertainty indicators were acceptable, suggesting that more than 70% of the observed data were bracketed by the 95% prediction uncertainty band. The validation results improved for both NS and R², which demonstrates a strong relationship between the simulated and observed flows. The overall performance of both models confirms that both were calibrated successfully and capable of accurately representing the hydrological behavior of the watershed.

**Table 5:** Statistical results for calibration and validation of the models.

Parameter	ILRI LULC-2004		Classified LULC-2021	
	Calibration	Validation	Calibration	Validation
NS	0.79	0.91	0.79	0.90
R^2^	0.79	0.96	0.79	0.94
p-factor	0.70	075	0.70	0.67
r-factor	1.32	0.59	1.30	0.57

**Fig. 8:**
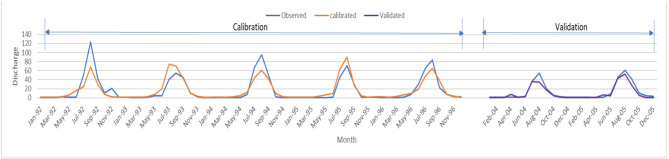
Time series of simulated and observed monthly stream flow for the calibration and Validation period for the classified LULC-2021 model at the Ribb station.

**Fig. 9:**
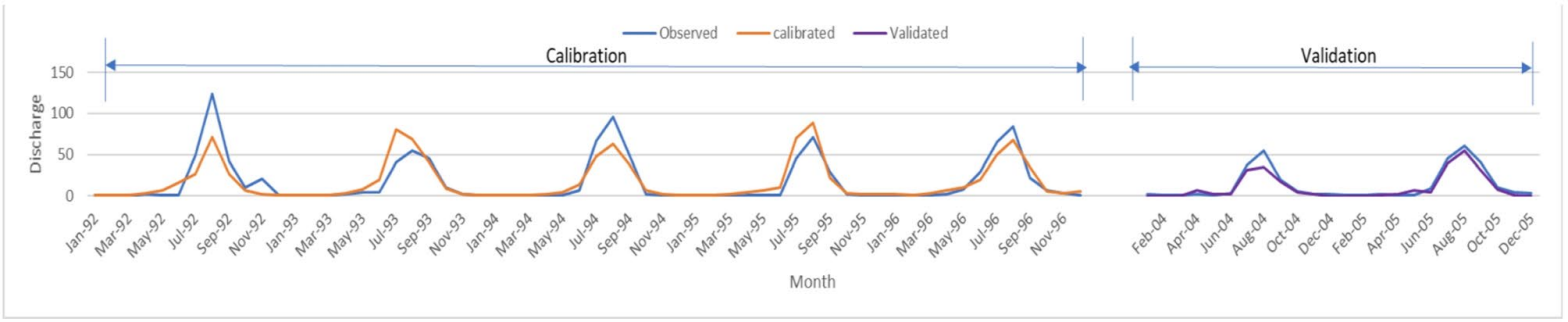
Time series of simulated and observed monthly stream flow for the calibration and Validation period for the ILIR LULC-2004 model at the Ribb station.

The calibration and validation results for streamflow at Ribb station are illustrated in Figs. 8 and 9. During the calibration period (1992–1996), the simulated discharge closely follows the observed hydrograph, capturing both peak and base flows with good agreement. The model slightly underestimates some peak flows, particularly in 1992 and 1995, overall, both models produces the seasonal dynamics well. In the validation period (2004–2005), the agreement between observed and simulated flows remains strong, with the hydrographs showing similar timing and magnitude of flow events. The gap between 1997 and 2003 represents a period with no observed data, and is intentionally shown as a break in the time series to emphasize the two distinct evaluation intervals. These graphical results agree with the statistical performance measures, confirming that the SWAT model is well calibrated and capable of reliably simulating streamflow in the Lake Tana Basin.

### Water balance investigation

The water balance was investigated for two distinct LULC scenarios—ILRI LULC-2004 and the newly developed classified LULC-2021 as shown in Table 6. The comparison shows the effects of changes in land cover on the hydrological processes of the basin. Both models provided balanced water budgets and showed almost identical annual precipitation values of 1609.1 mm/year for 2004 and 1608.3 mm/year for 2021 which is understandable because the same weather data were used for both models. The negligible change shows that the component changes were due to LULC changes rather than climatic variations. The surface runoff increased from 111.56 mm/year in 2004 to 118 mm/year in 2021 approximately 5.8%. However, the lateral flow decreased from 106.34 mm/year in 2004 to 100.72 mm/year in 2021. Shallow groundwater return flow, deep groundwater discharge, and deep aquifer recharge experienced minor changes confirming the minimal impact of LULC changes on deep surface hydrological processes. Evapotranspiration remained unaffected at 1066.7 mm/year in 2004 and 1066.2 mm/year in 2021 because the main source of evaporation was the large surface area of the lake itself with the large area of 3000 km^2^. Overall, the comparison revealed that the primary hydrological impact of land use changes between 2004 and 2021 was an increase in surface runoff and minor reductions in subsurface lateral flows. The results indicate the sensitivity of surface hydrological responses to LULC changes in the basin.

**Table 6:** Average annual water balance component from the calibrated SWAT models.

Hydrological parameter	ILRI LULC-2004 (mm/year)	Classified LULC-2021 (mm/year)
Precipitation	1609.1	1608.3
Surface runoff	111.56	118
Lateral flow	106.34	100.72
Shallow groundwater return flow	285.95	284.11
Deep groundwater discharge	19.55	19.22
Shallow aquifer evaporating	30.66	27.54
Deep aquifer recharge	19.27	19.27
Percolation out of soil	388.75	385.32
Evapotranspiration	1066.7	1066.2

### Discussion

The results of this study show that land use/cover (LULC) changes between 2004 and 2021 significantly influenced the hydrological processes of the Lake Tana Basin. The classified LULC- 2021 achieved high accuracy, which increases confidence in the detected changes. Forest and agricultural land showed notable declines (− 33% and − 10.2%, respectively), while wetlands (+ 81.4%) and rangelands (+ 299.2%) expanded considerably, with urban land emerging as a new class. This issue requires further investigation, as the continuation of these LULC change trends may potentially intensify surface runoff, reduce infiltration, and decrease groundwater recharge in the future, which could increase flood risks and threaten water availability in the Lake Tana Basin. Similar studies (e.g., Getachew et al., 2022; Engdaw et al., 2024) also reported the increase of runoff and reduced baseflow, supporting the findings of this study.

Despite these contributions, the study has two main limitations. First, the differences in hydrological responses between the ILRI-2004 and Landsat-2021 scenarios are partly attributed to their contrasting spatial resolutions; the ILRI map (~ 670 m) tends to smooth spatial variability and dampen model sensitivity, whereas the finer 30 m Landsat classification captures more detailed soil–plant–water interactions. The ILRI-2004 dataset lacks a reported accuracy assessment, which may partly contribute to the observed differences with the 2021 classification in addition to genuine LULC changes. Second, only two scenarios (2004 and 2021) were analyzed. Although additional Landsat-based classifications could be developed, preparing a third model and conducting full calibration and validation would require considerable time and is beyond the scope of this study. Future research should therefore consider multi-decadal Landsat image series at 10-year intervals to provide a more comprehensive assessment of LULC–hydrology interactions in the Lake Tana Basin. Such an approach could also reveal gradual long-term trends or potential tipping points in the basin’s hydrological response that are not captured in a two-point comparison.

Overall, the study demonstrates that LULC changes significantly affect the hydrological balance of the Lake Tana Basin, particularly by increasing surface runoff and reducing lateral flows. The results emphasize the importance of integrating sustainable land and water management strategies into basin planning to mitigate the effects of deforestation, rangeland expansion, and urbanization. Future research should extend the temporal analysis by incorporating multi-decadal Landsat series at 10-year intervals and include additional ground-truth validation to further improve classification accuracy and model reliability.

## Conclusions

The current study investigates the impact of land use / cover changes on the water balance of the Lake Tana basin using two maps; ILRI LULC-2004, and the classified LULC-2021, which was processed via the ERDAS imagine software. The classified LULC-2021 reached an overall accuracy of 91.11% and a Cohen’s kappa coefficient of 0.8937, which indicates almost perfect classification confirming the reliability of the classification. Comparisons between 2004 and 2021 revealed a 33% decline in forest cover and a 10.2% reduction in agricultural land, while rangelands (+ 299.2%) and wetlands (+ 81.4%) expanded considerably. Urban land was emerged as a new class in 2021. The ILRI LULC-2004 neglected urban areas, whereas the new map considered urban land use.

Land use changes between the classified LULC-2021 and the ILRI LULC-2004 impacted the relative sensitivity of subsurface and plant-interactive parameters. The classified LULC-2021 was more effective in soil–plant–water interactions which made parameters such as SOL_K, EPCO, and ALPHA_BF more pertinent. The ILRI LULC-2004 dampened the model’s sensitivity because of its lower resolution or more generalized land classification.

Additionally, an investigation of the water balance for both models revealed that both models were calibrated and validated and yielded acceptable results and balanced water budgets. The surface runoff increased by 5.8% (111.6 → 118 mm/year), while lateral flow decreased (106.3 → 100.7 mm/year). Evapotranspiration remained nearly constant (~ 1066 mm/year), largely driven by lake evaporation. Overall, the results emphasize the effects of land use land cover on surface runoff and lateral flow. More broadly, the current study demonstrates the importance of employing high-resolution and consistently classified LULC datasets to ensure reliable hydrological modeling and to capture the true impacts of land use/cover change on water processes. These findings ensure the importance of sustainable land and water management strategies to ensure long-term hydrological stability and water security in the basin.

## Data Availability

Any data can be obtained upon request by sending an email to the Correspondence author: es-EmanRagab2024@alexu.edu.eg. The collected data are available at the following links: https://www.earthdata.nasa.gov/. https://www.fao.org/. https://power.larc.nasa.gov/data-access-viewer/. http://ilri.org/.

## References

[1] Kebede, S., Travi, Y., Alemayehu, T. & Marc, V. Water balance of lake Tana and its sensitivity to fluctuations in rainfall, blue nile basin. *Ethiopia J. Hydrol.* **316**, 233–247 (2006).

[2] Setegn, S. G., Srinivasan, R. & Dargahi, B. Hydrological modelling in the lake Tana Basin, Ethiopia using SWAT model. *Open. Hydrol. J.* **2**, 49–62 (2008).

[3] Gebreyohannes, T. A. Climate change impact on hydrology of lake Tana Basin, upper blue nile Basin, Ethiopia. *J. Hydrol.* **598**, 126389. 10.1016/j.jhydrol.2021.126389 (2021).

[4] Wubneh, M. A. Operational analysis of lake Tana under climate change, upper blue nile Basin, Ethiopia. *Sci. Afr.* **24**, e02217. 10.1016/j.sciaf.2024.e02217 (2024).

[5] Mekonnen, D. F., Duan, Z., Rientjes, T. & Disse, M. Analysis of combined and isolated effects of land-use and land-cover changes and climate change on the upper blue nile river basin’s streamflow model. *Hydrol. Earth Syst. Sci.* **22**, 6187–6207 (2018).

[6] Kidane, M., Tolessa, T., Bezie, A. & Kessete, N. Evaluating the impacts of climate and land use/land cover (LU/LC) dynamics on the hydrological responses of the upper blue nile in the central highlands of Ethiopia. *Spat. Inf. Res.* **26**, 151–167 (2018).

[7] Getachew, B. & Alemayehu, T. Impacts of land-use change on the hydrology of lake Tana Basin, upper blue nile river Basin, Ethiopia. *Glob Challenges*. **6**, 2200041. 10.1002/gch2.202200041 (2022). 10.1002/gch2.202200041 PMC9360345 35958827

[8] Engdaw, F. F. Land use/land cover dynamics in the Northern watershed of lake tana: implications for water quality. *Front. Environ. Sci.* **12**, 1426789. 10.3389/fenvs.2024.1426789 (2024).

[9] FAO & IHE Delft. *Water Accounting in the Nile River Basin* (FAO WaPOR Water Accounting Reports, 2020).

[10] Shahin, M. *Hydrology of the Nile Basin* (International Institute for Hydraulic and Environmental Engineering, 1985).

[11] Yilma, A. D. & Awulachew, S. B. Characterization and atlas of the Blue Nile Basin and its sub-basins. In *Conf. Pap. H042502, International Water Management Institute* (2009).

[12] U.S. Geological Survey. EarthExplorer. https://earthexplorer.usgs.gov/ (2024).

[13] Landis, J. R. & Koch, G. G. The measurement of observer agreement for categorical data. *Biometrics* **33**, 159–174. 10.2307/2529310 (1977). 843571

[14] National Aeronautics and Space Administration (NASA). Earthdata. https://www.earthdata.nasa.gov/ (2022).

[15] Food and Agriculture Organization of the United Nations (FAO). Harmonized World Soil Database v1.2. https://www.fao.org/ (2022).

[16] NASA POWER. Data Access Viewer. https://power.larc.nasa.gov/data-access-viewer/ (2023).

[17] International Livestock Research Institute (ILRI). GeoPortal. http://ilri.org/ (2021). PMC1539864 10495906

[18] Huber, H. Investigation of hydrologic response unit (HRU) discretization for erosion modeling in SWAT in Upper Blue Nile Basin. (Technische Universität München, 2015).

[19] Neitsch, S. L., Arnold, J. G., Kiniry, J. R., Williams, J. R. & Soil and water assessment tool theoretical documentation version 2009. Technical Report No. 406 (Texas Water Resources Institute, 2011).

